# In Vitro Study for Lipolysis of Soybean Oil, Pomegranate Oil, and Their Blended and Interesterified Oils under a pH-Stat Model and a Simulated Model of Small Intestinal Digestion

**DOI:** 10.3390/nu11030678

**Published:** 2019-03-21

**Authors:** Chenming Ji, Jung-Ah Shin, Soon Taek Hong, Ki-Teak Lee

**Affiliations:** 1 Department of Food Science and Technology, Chungnam National University, 99 Daehak-ro, Yuseong-gu, Daejeon 34134, Korea; jcm0609@jsfpc.edu.cn (C.J.); jashin@cnu.ac.kr (J.-A.S.); hongst@cnu.ac.kr (S.T.H.); 2 Department of Food Science, Jiangsu Food & Pharmaceutical Science College, Huai’an 223003, China

**Keywords:** lipolysis, conjugated linolenic acid, triacylglycerol species, in vitro digestion model

## Abstract

In this study, two in vitro digestion models were employed to compare the rate of lipolysis in soybean oil (SBO), pomegranate oil (PGO), a physical blend (PHY, 1:1 molar ratio of SBO:PGO, *w*/*w*), and their enzymatically interesterified oil (IO). In the pH-stat digestion model (emulsified oils with bile salts), PGO emulsion containing 74.7% conjugated form of linolenic acid (CLn) showed a significantly lower release rate of free fatty acid (FFA) than the other oil emulsions (*p* < 0.05). In FFA release rates and oil droplet sizes between PHY and IO emulsions, no significant differences were observed (*p* > 0.05). In a simulated model of small intestinal digestion, the lipolysis rates of SBO, PGO, PHY, and IO after digestion for 30 min in digestion fluids were 80.4%, 66.5%, 74.8%, and 77.0%, respectively. The rate of lipolysis in PGO was significantly lower than that in SBO (*p* < 0.05), and the lowest lipolysis rate was observed in the conjugated form of trilinolenoyl glycerol (CLn-CLn-CLn).

## 1. Introduction

Pomegranate oil (PGO) is primarily composed of triacylglycerol (TAG) molecules with punicic acid (9Z,11E,13Z-octadeca-9,11,13-trienoic acid) in which double bonds are in a conjugated form [1]. The non-conjugated form, α-linolenic acid (9Z,12Z,15Z-octadeca-9,12,15-trienoic acid), is usually found in most vegetable oils [2,3]. Several studies have proposed that PGO containing high content of conjugated linolenic acid (CLn), such as punicic acid, may offer beneficial effects relating to inflammation and colon cancer [4,5]. However, the chemical nature of TAG in fats and oils can affect lipid bioavailability and digestibility. For example, it is known that both a shorter length and fewer double bonds in fatty acids promote the hydrolysis rate by pancreatic lipase [6,7]. Arishima et al. [8] also reported that the presence of behenic acid (C22:0) in TAG molecules slows a significant reduction of lipolysis rate. Further, in an in-vitro pancreatic lipase assay, more hydrolysis of non-conjugated linoleic acids (9Z,12Z-octadeca-9,12-dienoic acid) than that of the conjugated form of linoleic acids from TAG molecules has been observed [9]. Because pancreatic lipase is known to have *sn*-1,3 specificity relating to TAG molecules, TAG species that have different positional fatty acid compositions may induce different lipolysis rates. 

Interesterification is one possible approach to prepare the counterparts of simple oil blends, in which oils have similar total fatty acid compositions but different TAG species. During the interesterification reaction, fatty acids located in TAG molecules are exchanged, causing rearrangement of fatty acids on the glycerol backbone until a thermodynamic equilibrium is reached [10,11,12,13].

A pH-stat titration system has been used to evaluate lipolysis rates of lipids by in vitro digestion [14,15,16]. In this system, the lipolysis rate is measured by determining the amount of released fatty acids induced by pancreatic lipase from substrates, which are neutralized by alkali titration.

Very few studies to date have used high-performance liquid chromatography (HPLC) analysis to measure the degree of lipolysis that occurs in in vitro digestion models. For example, Giang et al. [17] quantified the remaining TAG in masses after the lipolysis using HPLC. Despite the possible beneficial physiological functions of PGO as noted above, experiments assessing in vitro digestion models have been limited.

In this study, a physical oil blend (PHY) and an interesterified oil (IO) were prepared from soybean oil (SBO) and PGO. Thus, PHY and IO would have similar total fatty acid compositions while having different TAG species. Emulsifications of four different oils (SBO, PGO, PHY, and IO) with bile salts were prepared, and the particle sizes and physical stabilities of lipid droplets were compared. Further, the effect of lipolysis on TAG molecules with different compositions was studied using a pH-stat digestion model. Meanwhile, a model that simulated small intestinal digestion was used along with HPLC analysis to evaluate quantitative changes in individual TAG species after lipolysis of oils in digestion fluids with bile extracts. 

## 2. Materials and Methods

### 2.1. Materials

Soybean oil (SBO) and pomegranate oil (PGO) were provided by CJ-Cheil-Jedang Co. (Seoul, Korea) and Kerfoot Inc. (North Yorkshire, UK), respectively. Immobilized lipase from *Rhizomucor miehei* (Lipozyme^®^ RMIM) was obtained from Novozymes Korea (Seoul, Korea). Pancreatin (P3293) and type II lipase (L3126) from porcine pancreases, bile salts (B8756), porcine bile extract (B8631), and bovine serum albumin (BSA, A7906) were obtained from Sigma-Aldrich Korea (Yongin, Korea). Triundecanoin was purchased from Nu-Chek-Prep (Waterville, ME, USA). Sodium chloride (NaCl), sodium bicarbonate (NaHCO_3_), monopotassium phosphate (KH_2_PO_4_), potassium chloride (KCl), magnesium chloride (MgCl_2_), calcium chloride (CaCl_2_), hydrogen chloride (HCl) and sodium hydroxide solution (0.05 N, NaOH) were purchased from Daejung Chemicals & Metals Co. (Siheung, Korea). Urea was purchased from Junsei Chemical Co. (Tokyo, Japan). The solvents used for HPLC and gas chromatography (GC) analysis were purchased from Fisher Scientific Korea (Seoul, Korea).

### 2.2. Preparation of Oil Samples

Interesterified oils (IO) and a physical blend (PHY) were prepared with SBO and PGO. IO and PHY contained similar total fatty acid compositions but different TAG species profiles. To prepare IO, SBO and PGO (1:1 molar ratio, *w*/*w*) were mixed with Lipozyme^®^ RMIM (20% by total weight of substrates). Interesterification was carried out for 6 h at 50 °C in a water bath shaker at 200 rpm. PHY was prepared by simple blending with SBO and PGO at molar ratio of 1:1 (*w*/*w*).

Subsequently, SBO, PGO, IO, and PHY were passed through a column to separate TAG from free fatty acids (FFAs), diacylglycerol (DAG) and monoacylglycerol (MAG). Briefly, each oil (20 g) was dissolved in *n*-hexane (10 mL) and was applied to the column (3.5 cm diameter, 20 cm length) packed with silicic acid (20 g), and then eluted with 250 mL *n*-hexane. If necessary, this step was performed repeatedly until isolation of TAG was confirmed by thin-layer chromatography, where the absence of DAG, MAG, and FFA bands was confirmed [18]. A vacuum rotary evaporator was used to remove the *n*-hexane at 40 °C, and traces of *n*-hexane were removed by flushing with nitrogen. Each obtained sample was stored at −30 °C until further analysis.

### 2.3. Total Fatty Acid Composition Analysis

The total fatty acid composition of the samples was analyzed by GC after methylation [19]. Analysis was performed in duplicate, and the results are expressed as the average value and standard deviation. An Agilent 6890 Gas Chromatograph (Santa Clara, CA, USA) equipped with an auto-injector and a flame-ionization detector was used for fatty acid composition analysis. The column (SP^TM^-2560, 100 m × 0.25 mm i.d., 0.2 µm film thickness; Supelco, Bellefonte, PA, USA) was maintained at 150 °C for 5 min, increased to 220 °C at a rate of 4 °C/min, and then held at 220 °C for 30 min. The carrier gas was helium with a flow rate of 1 mL/min in constant flow mode. The spilt ratio was 50:1. The injector and detector temperatures were 250 °C and 260 °C, respectively. The retention time of each fatty acid was compared with that of the Supelco 37 component FAME mixture (10 mg/mL, Sigma-Aldrich/Merck KGaA, Darmstadt, Germany).

### 2.4. Preparation of Digestion Fluids

The compositions of duodenal juice and bile juice are described in Table 1, which was prepared based on previous works with slight modifications [20,21].

### 2.5. Particle Size and Physical Stability of Emulsified Oils in Digestive Fluid

Each emulsion was prepared by dispersing 83 mg of oil (SBO, PGO, PHY, and IO) into 10 mL of freshly prepared digestion fluid [a mixture of duodenal juice and bile juice (2:1, *v*/*v*)] via an ultrasonic processor (VC750, Sonics & Materials Inc., Newtown, CT, USA) in a 25-mL glass vial for 1 min (6200–6300 Joules), respectively. The oil droplet size was measured by a laser diffraction analyser (Mastersizer S, Malvern Instrument Ltd., Worcestershire, UK). The particle size was reported as the surface-weighted mean diameter, d_32_ = ∑n_i_d_i_^3^/∑n_i_d_i_^2^, where n_i_ is the number of particles with diameter (d_i_). Samples were analyzed in duplicate.

The physical stability of an oil droplet in digestion fluid was analyzed using a Turbiscan Lab Expert (Toulouse, France). The prepared emulsion was transferred into a cylindrical glass cell and scanned by the detection head of the Turbiscan, which consisted of one pulsed near-infrared light source (λ = 880 nm) and two synchronous detectors: a transmission (T) and a back scattering (BS) detector. The detection head scanned the total height of the sample (23–24 mm) every 5 min for 3 h at 25 °C. The region from the liquid level to 3 mm below was set as the creaming layer zone, and that from the bottom to 5 mm above was set as the clarification layer zone. The data were collected and analyzed by Turbiscan Lab Expert software.

### 2.6. Lipolysis in a pH-Stat Digestion Model

To study the lipolysis rate in each emulsion, a pH-stat digestion model was employed to simulate small intestinal digestion in an in vitro system. Digestion fluid was freshly prepared by mixing duodenal juice and bile juice (2:1, *v*/*v*) before the experiment. Each oil sample (approximately 300 mg) was dispersed into 35 mL of digestion fluid in a beaker (100 mL) by an ultrasonic processor (VC750, Sonics & Materials Inc., Newtown, CT, USA) for 1 min (6200–6300 Joules). A potentiometric automatic titration (AT-400E, Kyoto Electronics Manufacturing Co., Tokyo, Japan) was applied to monitor the FFA release rate. The freshly prepared digestion fluid was kept at 37 °C. The lipolysis process was started by adding an additional 1 mL digestion fluid containing 106 mg of pancreatin and 71 mg of type II lipase into the digestion cell (i.e., a 100 mL beaker). The digestion cell was kept at 37 °C in a water bath. A magnetic bar was used for gentle mixing (150 rpm). Thus, the final concentrations were approximately 8.3 mg oil/mL digestion fluid, 1.0% bile salts, 0.25% BSA, 3 mg/mL pancreatin, 2 mg/mL type II lipase, 216 mM NaCl, 13.6 mM KCl, 97 mM NaHCO_3_, 2.8 mM CaCl_2_, 0.8 mM KH_2_PO_4_, 0.7 mM MgCl_2_, and 4.9 mM urea in a total volume of 36 mL of digestion fluids. During digestion, the pH of the titration was automatically maintained at 8.1 by a 0.05 N NaOH solution. The consumed volume of NaOH solution was recorded for the calculation of the released FFA percentage according to a previous report [14].

$$\mathrm{FFA}\;\left(\%\right)=\frac{V_{\mathrm{NaOH}\left(t\right)}\times m_{\mathrm{NaOH}}}{w_{\mathrm{lipid}}/M_{\mathrm{lipid}}\times 2}\times 100$$

where V_NaOH(t)_ is the consumed volume (L) of NaOH solution applied to maintain pH 8.1 of the digestion system at time t, m_NaOH_ is the molarity (mol/L) of the employed NaOH solution, w_lipid_ is the weight (g) of the oil sample in the digestion system at 0 min, and M_lipid_ is the molecular weight (g/mol) of the oil sample. Duplicate analysis was performed. The initial rate was defined as the number of micromoles of FFA leaving the droplets per 1 min of the lipolysis reaction.

$$\mathrm{Initial}\;\mathrm{rate}\;\left(\mathrm{mM}/s\right)=\frac{\mathrm{Released}\;\mathrm{FFA}\;\mathrm{amount}\;\left(µ\mathrm{mol}\right)\;\mathrm{during}\;1\min\mathrm{reaction}\;}{\mathrm{Total}\;\mathrm{volumn}\;\left(36\;\mathrm{mL}\right)}÷60\;\left(\sec\right)$$

### 2.7. Lipolysis in a Model that Simulated Small Intestinal Digestion

In this study, bile salts were replaced by bile extract to prepare the digestion fluid. The bile extract was filtered to remove insoluble compounds. Each oil sample (approximately 150 mg) was mixed with digestion fluid (17 mL) in a conical flask (250 mL) with a screw cap. The flask was placed in a water bath shaker for 2 min at a speed of 60 rpm at 37 °C. Lipolysis was started by adding 1 mL of digestion fluid into the conical flask that contained 53 mg of pancreatin and 35.5 mg of type II lipase. At 0 min (i.e., before the enzyme was added) and 30 min after lipolysis, the sample was poured into a 50 mL cylindrical glass tube. Diethyl ether (10 mL) was then added to extract the lipolysis products. After shaking vigorously for 1 min, centrifugal separation (15 min, 2500× *g*) was performed to obtain the upper layer, which was subsequently applied to an anhydrous sodium sulfate column to remove residual moistures. Extraction was repeated 3 times. After collecting the upper layer into a new glass tube, diethyl ether was completely removed by nitrogen evaporation. The extracted lipolysis product was then weighed and re-dissolved in 3 mL of diethyl ether. The obtained samples were stored at −20 °C until HPLC analysis.

### 2.8. HPLC Analysis

For HPLC analysis, lipolysis products in diethyl ether were transferred to a glass tube, and diethyl ether was evaporated by nitrogen. The dried lipolysis products were then re-dissolved in acetone containing triundecanoin as an internal standard (IS) to adjust the mixture to a final concentration of 1 mg lipolysis product/mL and 0.05 mg triundecanoin/mL. The sample was analyzed by HPLC according to methods outlined previously [22] with slight modifications. The analysis of TAG species was performed by a Yonglin SP930D HPLC (Yonglin, Anyang, Korea) connected to a Sedex 75 evaporative light-scattering detector (ELSD, Sedere, Alfortville, France). The nebulizing temperature and nitrogen gas were kept at 40 °C and at pressure of 2.2 bars, respectively. A Nova-Pak^®^ C18 column (150 × 3.9 mm, 4 µm, Waters, Milford, Ireland) was used. Separation was achieved by a gradient elution that consisted of acetonitrile (A) and isopropanol/*n*-hexane (2:1, *v*/*v*) (B) administered at a flow rate of 1 mL/min with the following elution protocol (elapsed time, ratio of solvent B): 0 min, 20% B; 45 min, 46% B; 60 min, 46% B; 65 min, 20% B; 70 min, 20% B. TAG species were designated by comparing retention times and partition numbers (PN) with those of various TAG standards obtained from Nu-Chek-Prep (Waterville, ME, USA), where the latter was calculated using the following equation: PN = total carbon number (CN) − 2 × total number of double bonds (ND). Based on fatty acid composition, PN, and previous reports [23,24], plausible TAG species were also designated. For quantification of TAG molecules to calculate the lipolysis rate, the concentrations of SBO at 0.3–1.2 mg/mL, PGO at 0.2–1.0 mg/mL, physical blend at 0.2–1.0 mg/mL, and IO at 0.2–1.0 mg/mL were injected into HPLC system. All analyses were carried out in duplicate. To determine the lipolysis rate of each oil sample, the total areas of TAG peaks at 0 and 30 min were obtained from the HPLC chromatogram, and the areas of the IS peak at 0 and 30 min were obtained simultaneously. Calibration curves assessing the total peak area ratio of TAG/IS versus the concentration ratio of oil/IS were then obtained by applying the following the power models: y = 0.4568x^1.4353^, R^2^ = 0.996 (SBO); y = 0.9241x^1.4759^, R^2^ = 0.952 (PGO); y = 0.32x^1.5251^, R^2^ = 0.994 (PHY), and y = 0.4538x^1.4306^, R^2^ = 0.999 (IO).

### 2.9. Statistical Analysis

Statistical analysis was performed with SPSS 16.0 software (SPSS, Inc., New York, NY, USA). Duncan’s multiple-range test was applied to determine significant differences among test values (*p* < 0.05 and *p* < 0.1 were considered as significant differences).

## 3. Results

### 3.1. Characteristics of Oil Samples

In SBO, linoleic acid (L, 49.5%) and oleic acid (O, 22.1%) are major compositional fatty acids, while pucinic acid (CLn, 74.7%) is the most abundant fatty acid in PGO, in accordance with the previous report [1]. As expected, the analyzed fatty acid composition of PHY was close to the theoretical value of the blend mixture (SBO and PGO at a molar ratio of 1:1, *w*/*w*). Total fatty acid composition of IO was not much different from that of PHY, which contained 32.9 area% linoleic acid, 29.2 area% pucinic acid, and 16.7 area% oleic acid, indicating that the interesterification reaction did not markedly affect the fatty acid composition of IO (Table 2). However, the interesterification reaction induced rearrangement of fatty acids on the glycerol backbone of TAG molecules [10,11,12,13]. For example, the major components of TAG species in PGO were 67.8% TAG with PN = 36 (CLn-CLn-CLn) and 12.1% TAG with PN = 40 (CLn-CLn-O, CLn-CLn-P, etc.). PHY was composed of CLn-CLn-CLn (24.8 area%) along with L-L-L and L-L-O that originated from SBO. After the enzymatic interesterification reaction, however, TAG with PN = 38 increased to 18.0 area% in IO, which was observed as 3.0 area% in PHY. This result suggests that some CLn-CLn-CLn (PN = 36) was converted to TAG that exhibits a PN = 38 (possibly corresponding to CLn-CLn-L and CLn-Ln-L) because TAG with PN = 36 (CLn-CLn-CLn, CLn-CLn-Ln, etc.) decreased to 3.4 area% in IO. Similarly, L-L-L (PN = 42) composed of 12.9 area% in PHY, which decreased to 2.2 area% in IO, indicating that the interesterification reaction induced the formation of newly re-structured TAG molecules in IO (Table 2).

### 3.2. Particle Size and Physical Stability of the Emulsified Oils in Digestive Fluids

**Figure 1:**
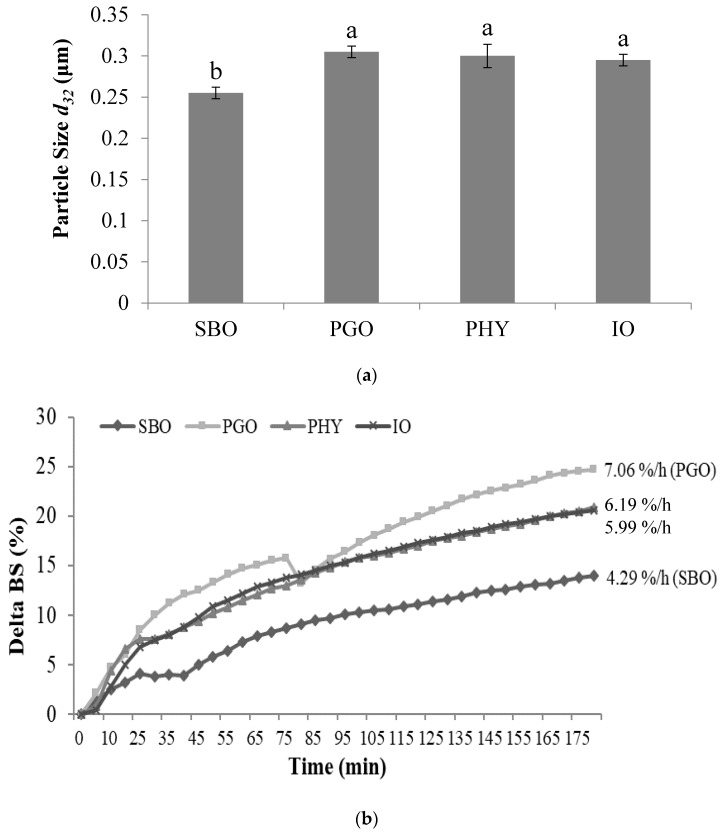

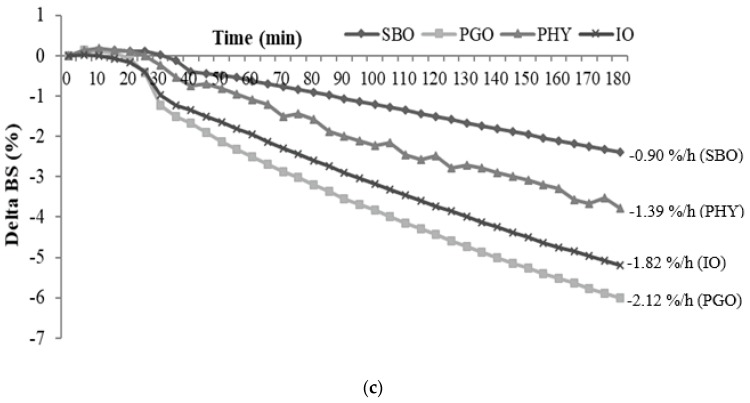
The mean particle sizes (*d*_32_, μm) (**a**); delta back scattering (ΔBS) values (%) and ΔBS(t) rates (%/h) of creaming layer (**b**); and clarification layer (**c**) of the emulsified oils in digestion fluid. Statistical differences are indicated by different letters (**a**,**b**) where significant differences were detected (*p* < 0.05). Abbreviation: SBO, soybean oil; PGO, pomegranate seed oil; PHY, physical blend; IO, interesterified oil.

Droplet size is one of the most important factors affecting the lipid digestion rate since the available surface area of the lipid droplet where the lipases bind increases as the droplet particle size decreases [25,26]. Therefore, droplet sizes (*d*_32_) were measured (Figure 1a). The *d*_32_ values of the emulsified SBO, PGO, PHY, and IO in digestion fluid were 0.26, 0.31, 0.30, and 0.30 μm, respectively. The statistical analysis showed that the *d*_32_ of the emulsified SBO was significantly smaller than that of the others (*p* < 0.05). Smaller droplet size of lipid globules in SBO emulsion may offer a larger available surface area for lipase binding, and thus faster lipolysis was expected in SBO emulsion than with the other samples (PGO, PHY, and IO emulsion). This result may be due to different TAG species and a different fatty acid composition (Table 2). In addition to droplet size, the physical stability of the lipid droplets in digestion fluid was monitored using a Turbiscan Lab Expert. Detection was performed to monitor the change in the delta backscattering (ΔBS, %) value that represents the stability of lipid droplets in digestion fluid [27,28]. Figure 1b shows a ΔBS (%) change in the 3 mm region from samples (namely, creaming layer zone). The increase of ΔBS (%) during the measurement time (180 min) suggested that the creaming layer gradually formed, where lipid droplets were progressively concentrated [29]. Overall, ΔBS (%) level of the emulsified SBO was the lowest, while that observed in the emulsified PGO was the highest. As shown in Figure 1b, PGO emulsion exhibited a marked increase in ΔBS (%) during the analysis time, showing the highest ΔBS(t) rate (7.06 %/h) in the creaming layer zone, while SBO emulsion showed the lowest (4.29%/h). In the case of the emulsified PHY and IO samples, the differences in ΔBS (%) were not notable between them, and the ΔBS (%) of PHY and IO emulsions were observed between those in SBO and PGO emulsions during the determination time. The ΔBS (%) of the clarification zone (5 mm region from the bottom to up) is presented in Figure 1c, where the ΔBS(t) rate (%/h) decreased in the order of SBO (−0.90) > PHY (−1.39) > IO (−1.82) > PGO (−2.12%/h), suggesting that the lipid droplets from the emulsified SBO in digestion fluids were the most stable, while the droplets from the emulsified PGO were the least stable. Such results agree with those found during lipid droplet measurement (Figure 1a).

### 3.3. The Effect of Lipid Composition on Lipolysis Using a pH-Stat Model

**Figure 2:**
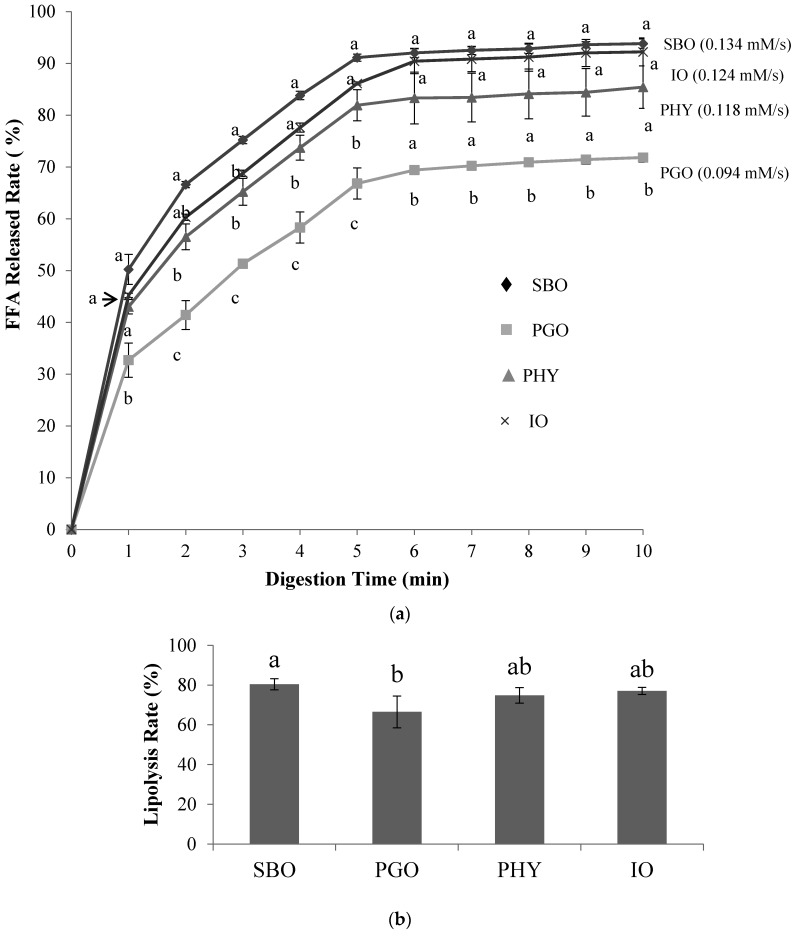
The free fatty acid (FFA) release rates (%) and initial rates (mM/s) in a pH-stat digestion model (**a**); and lipolysis rates (%) after 30 min of digestion in the simulated model of small intestinal digestion (**b**). Statistical differences are indicated by letters where significant differences were detected (*p* < 0.05). Abbreviation: SBO, soybean oil; PGO, pomegranate seed oil; PHY, physical blend; IO, interesterified oil.

SBO is widely-consumed as an edible oil containing C18:2 (L, n-6) and C18:3 (Ln, n-3) known as essential fatty acids, whereas PGO contains a high amount of unusual conjugated linolenic acid (CLn) such as pucinic acid (Table 2). Meanwhile, PHY and IO were prepared from SBO and PGO, which have similar compositions of total fatty acids but have different TAG species profiles. These four samples were used to study the effects of lipid composition on lipolysis. In Figure 2a, lipolysis of all samples was slower after 5–6 min because the lipolysis products could compete with lipase at the interface of the lipid droplets, which slowed the lipolysis rate even though bile salts can promote lipase adsorption by removing lipolysis products (FFAs, MAGs, etc.) from the interface of the lipid droplet [26,30]. Moreover, the FFA release rates (%) of SBO (93.8%), IO (92.2%), and PHY emulsions (85.4%) at 10 min were significantly different from that observed in the PGO emulsion (71.8%) (*p* < 0.05). This might be partly due to the existence of the conjugated form of fatty acids (CLn) in PGO. A previous report suggests that the release of conjugated linoleic acid is lower than that in non-conjugated linoleic acid in in vitro digestion models [9]. In addition, PHY and IO emulsions did not show a significant difference in their degree of lipolysis (*p* > 0.05) at 10 min, even though a slightly reduced FFA release rate was observed in the PHY emulsion during the lipolysis reaction. However, the emulsified PHY and IO samples showed higher FFA release rates (%) than the PGO emulsion (*p <* 0.05). Consequently, the initial lipolysis rate of the emulsified PGO (0.094 mM/s) was significantly lower than those of SBO (0.134 mM/s), PHY (0.118 mM/s), and IO (0.124 mM/s) emulsions (*p* < 0.05). These results suggest that the reduced content of CLn in PHY and IO increased lipolysis rates, while different TAG species in the PHY and IO profiles did not affect lipolysis rates much in these experimental conditions. Based on these results, the rate of lipolysis might be affected by the fatty acid composition of TAG molecules as well as by emulsion stability and lipid droplet sizes, as previously reported [14,31,32].

### 3.4. Lipolysis in a Simulated Model of Small Intestinal Digestion

An HPLC-ELSD instrumental analysis was employed to study the lipolysis rate of SBO, PGO, PHY, and IO in digestion fluids after 30 min of digestion in a simulated model of small intestinal digestion. 

Lipolysis rates were obtained based on the assumptions described below because ELSD often provides a power function equation for the calibration curve as suggested [33,34,35]. Calibration curve can obtain from concentration (mg/mL) of injected lipid versus detector response (area) of ELSD. The peak area from ELSD would be considered as A = aC^b^, where A is the peak area of sample, C is the sample concentration, and a and b are coefficients. In this experiment, triundecanoin was used as an internal standard (IS). Hence, the power function equation was converted to

(1) $$A_{\mathrm{TTGS}}/A_{\mathrm{IS}}=a{\left(C_{\mathrm{TTGS}}/C_{\mathrm{IS}}\right)}^{b}$$

where A_TTGS_/A_IS_ is the area ratio between total TAG species (TTGS) in each oil and IS, and C_TTGS_/C_IS_ is the concentration ratio between them. 

The C_TTGS_ of the lipolysis product can then be calculated by the calibration curve equation. Based on the known concentration of the injected sample (C_S_, 1 mg/mL) and weight of lipolysis product (m_LP_), the mass of TTGS (m_TTGS_) in lipolysis product could be calculated as

(2) mTTGS=[(ATTGS/AIS)/a]1bCISmLP/CS

To normalize the amount of oil sample (150 mg) used in the lipolysis process, Equation (2) was modified to

(3) mTTGS=[(ATTGS/AIS)/a]1bCISmLP/CSma×150

Here, m_a_ is the actual amount of oil sample used in lipolysis process.

Meanwhile, the lipolysis rate of oil sample after t min of the lipolysis reaction can be expressed by TTGS as the amount of lipolysis product at 0 and t min as

(4) $${\mathrm{Lipolysis}\;\mathrm{rate}}_{t}\;\%=\frac{m_{\mathrm{TTGS}\left(0\right)}-m_{\mathrm{TTGS}\left(t\right)}}{m_{\mathrm{TTGS}\left(0\right)}}\times 100$$

Substituting Equation (3) into Equation (4) leads to

(5) $${\mathrm{Lipolysis}\;\mathrm{rate}}_{t}\;\%=\left(1-\frac{{\left(A_{\mathrm{TTGS}\left(t\right)}A_{\mathrm{IS}\left(0\right)}\right)}^{1/b}m_{\mathrm{LP}\left(t\right)}m_{a\left(0\right)}}{{\left(A_{\mathrm{TTGS}\left(0\right)}A_{\mathrm{IS}\left(t\right)}\right)}^{1/b}m_{\mathrm{LP}\left(0\right)}m_{a\left(t\right)}}\right)\times 100$$

Therefore, the coefficient “b” can be determined by curve fitting and relating the peak area ratio of total TAG/IS to the concentration ratio of oil/IS where the lipolysis rate can then be obtained.

As shown in Figure 2b, the lipolysis rates (%) of SBO, PGO, PHY, and IO in digestion fluids after 30 min of digestion were 80.4%, 66.5%, 74.8%, and 77.0%, respectively. The lipolysis rate of the SBO in digestion fluids was significantly higher than that of the PGO (*p <* 0.05), while no significant difference was observed among lipolysis rates of the SBO, PHY, and IO samples (*p* > 0.05). However, a slightly higher rate of lipolysis rate could be observed in the SBO sample compared to the PHY and IO samples. Such results show a similar trend to those from the pH-stat digestion system, where the lipolysis rate was in the following order: SBO ≥ IO ≥ PHY > PGO.

**Figure 3:**
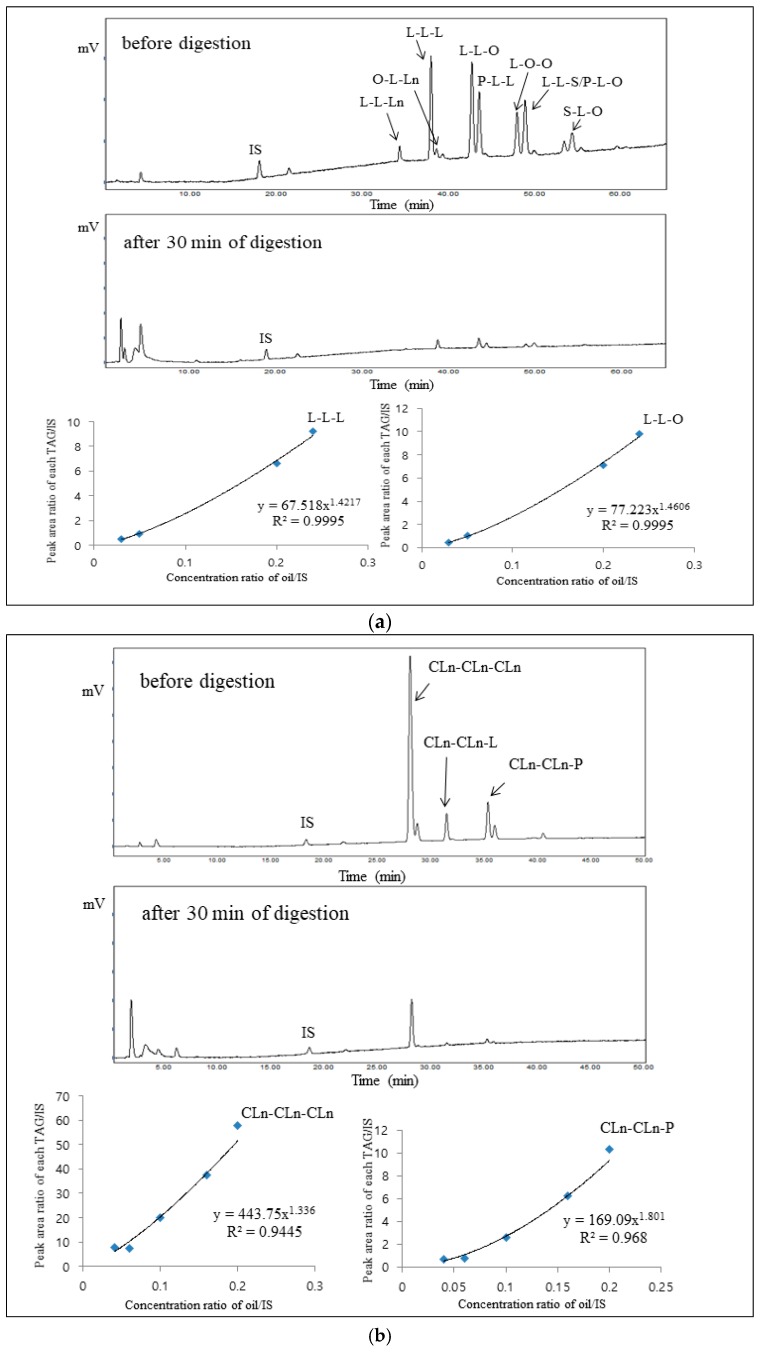
HPLC Chromatograms of soybean oil (**a**) and pomegranate seed oil (**b**) before digestion and after 30-min digestion. The calibration curves (concentration ratio of soybean oil/internal standard versus the peak area ratio of individual triacylglycerol species/internal standard) of trilinoleoyl glycerol (L-L-L), dilinoleoyl-oleoyl glycerol (L-L-O), triconjugated linoleonyl glycerol (CLn-CLn-CLn), and diconjugated linoleonyl-palmitoyl glycerol (CLn-CLn-P) triacylglycerol (TAG) species are shown. Abbreviation: O, oleic acid; L, linoleic acid. CLn, conjugated linolenic acid; P, palmitic acid.

**Figure 4:**
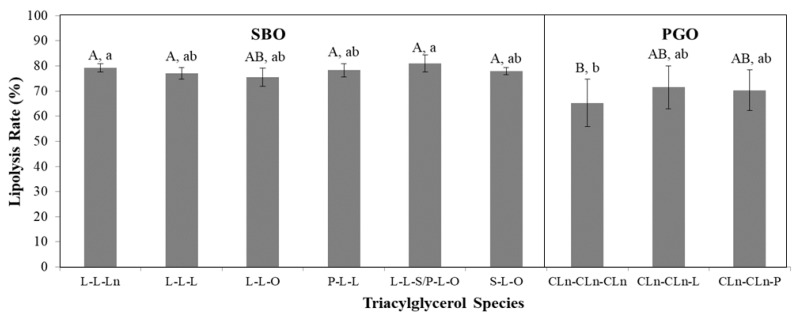
Lipolysis rates (%) of individual triacylglycerol (TAG) species in soybean oil (SBO) and pomegranate seed oil (PGO) after 30 min of digestion in the simulated model of small intestinal digestion. Statistical differences are indicated by letters where significant differences were detected (*p* < 0.1 for A and B; *p* < 0.05 for a and b). Abbreviation: P, palmitic acid; S, stearic acid; O, oleic acid; L, linoleic acid; Ln, linolenic acid; CLn, conjugated linolenic acid.

Furthermore, the lipolysis rates of the selected individual TAG species in either SBO or PGO were obtained. For calculating the lipolysis rate (%) of each TAG species, the area of each TAG species was substituted for A_TTGS_ in Equations (1)–(5). Therefore, based on the assumptions mentioned above, the calibration curves for the concentration ratio of oil/IS versus the peak area ratio of each TAG/IS were obtained by applying the power model. For the selected TAG peaks, the models proposed are as follows: y = 67.518x^1.4217^, R^2^ = 0.9995 (L-L-L); y = 77.223x^1.4606^, R^2^ = 0.9995 (L-L-O); y = 443.75x^1.336^, R^2^ = 0.9445 (CLn-CLn-CLn); and y = 169.09x^1.801^, R^2^ = 0.968 (CLn-CLn-P), respectively (Figure 3a,b). Chromatograms were obtained from the 30-min digestion of SBO (Figure 3a) and PGO (Figure 3b) using a model that simulates small intestinal digestion. After 30 min of digestion, the smaller peaks in SBO were observed than in PGO while a peak corresponding to CLn-CLn-CLn present in PGO was clearly visible. This result (Figure 3) can be further explained specifically from the following result (Figure 4) in which CLn-CLn-CLn showed the lowest lipolysis rate among TAG species in PGO and SBO.

In Figure 4, lipolysis rates (%) of the selected individual triacylglycerol (TAG) species after 30 min of digestion in a simulated model of small intestinal digestion were presented. Each individual TAG species in SBO sample was not significantly different in its lipolysis rate, where L-L-L (trilinoleoyl glycerol) and L-L-O (dilinoleoyl-oleoyl glycerol) presented as abundant TAG species that were similarly hydrolyzed to S-L-O (stereoyl-linoleoyl-oleoyl glycerol) that account for approximately 4.2% in SBO (Table 2). Overall, most TAG species in the SBO sample showed lipolysis rates ranging from 77.1% (L-L-L) to 81.0% (L-L-S/P-L-O) while those of CLn-CLn-CLn, CLn-CLn-L, and CLn-CLn-P in the PGO sample showed rates of 65.3%, 71.5%, and 70.3%, respectively. When compared to L-L-L of SBO, the lipolysis rate in CLn-CLn-CLn of the PGO was lowered by 11.8%. However, there was no significant difference in lipolysis rates between them at *p* = 0.05, but only at *p* < 0.1, CLn-CLn-CLn in PGO sample showed a significantly different lipolysis rate when compared to other TAG species in SBO sample except L-L-O (Figure 4). This study suggested that a simulated small intestinal digestion system along with HPLC instrumental analysis could evaluate not only the lipolysis rate of oil samples but also evaluate the quantitative changes in individual TAG species.

## 4. Discussion

It is expected that the lipolysis rate of TAG molecules varies depending on the fatty acid (saturation and unsaturation, carbon number, double bond number, distribution of fatty acids, etc.) [6,7,8]. As seen in the pH model (Figure 2a), the initial rate (mM/s) of IO was 0.124 mM/s, which is slightly faster than PHY (0.118 mM/s). Although the FFA release rate of IO at 10 min did not show a statistically significant difference compared with that of PHY, it was high with significant difference until the 5 min lipolysis. This may be attributable to the fat that CLn-CLn-CLn of PGO, which is not well lipolyzed, exists in IO as restructured TAG molecules, such as CLn-CLn-L after the interesterification reaction (Table 2). PGO used in this study consisted of 67.8% CLn-CLn-CLn. Although fatty acid compositions of PHY and IO were similar to each other, PHY was mainly composed of CLn-CLn-CLn (24.8%), along with L-L-L and L-L-O, which originated in SBO. On the other hand, IO was consisted of at most 3.4% TAG molecules, possibly corresponding to CLn-CLn-CLn after enzymatic interesterification; this suggests that the restructured TAG molecules are synthesized due to this reaction [10,11,12,13]. From the results, we suggested that the amount and the location of CLn on the TAG molecules affect the lipolysis rate. In particular, the TAG consisting of three molecules of CLn (i.e., CLn-CLn-CLn) is considered to be the most important factor for low lipolysis in this study. Indeed, the length of carbon chain and the position of double bonds of fatty acid (i.e., the degree to which double bond is close to carboxyl group, and conjugated/non-conjugated form), in addition to their positional distribution on the TAG molecules, affect the lipolysis rate. The following previous studies supported this suggestion: the emulsified lipid consisting of short chain fatty acids showed higher lipolysis than that of long chain fatty acids [7]; fatty acids with a double bond located close to the carboxyl group tended to show low lipolysis rates, partly due to a steric hindrance during the substrate–enzyme complex formation [8,36,37]; conjugated linolenic acid showed a lower lipolysis rate than linoleic acid, and *cis* and *trans* form of double bond would have a certain effect on lipolysis rate [9]; and, when the positional distribution of fatty acid on the TAG molecules was changed, different lipolysis rates were observed, in which the lipolysis rates of S-O-O and O-S-O (i.e., TAG molecules with different stearic acid position) were 0.63 and 0.56 mM/h, respectively [8].

The droplet size is known to be one of the factors affecting the digestibility of lipids [7,25,26]. The droplet size (*d*_32_) of the SBO emulsion (0.26 μm) was significantly smaller than that observed in other emulsions, while *d*_32_ in PGO (0.31 μm), PHY (0.30 μm), and IO (0.30 μm) showed no significant difference to one another. Nevertheless, we considered that a droplet size difference of only 0.04–0.05 μm would have a less significant effect on lipolysis than the aforementioned CLn-CLn-CLn content. In a previous study, the lipolysis rate with a difference in droplet size was reported. Although lipolysis of the fine emulsion was higher than that of the coarse emulsion, the droplet sizes were 0.7 μm for fine and 10 μm for coarse emulsion, resulting in a very large difference in the droplet size than our experiment [25].

## 5. Conclusions

The results obtained from the pH-stat digestion model showed that the FFA release rate in the emulsified PGO that contains a high amount of CLn was significantly lower than the rates observed in SBO, PHY, and IO emulsions in these study conditions (*p <* 0.05). Although the *d*_32_ of the SBO emulsion was significantly smaller than that observed in the other emulsions and the ΔBS(t) rate decreased in the order of SBO (−0.9) > PHY (−1.39) > IO (−1.82) > PGO (−2.12%/h) emulsions, the lowest lipolysis rate from the PGO emulsion seems to be mainly due to the TAG species that are comprised of CLn-CLn-CLn. More specifically, the FFA release rates of PGO (71.8%) were significantly lower than those of SBO (93.8%), IO (92.2%), and PHY (85.4%), when the pH-stat digest model was performed for 10 min. Meanwhile, PHY and IO emulsions did not show a significant difference (*p* > 0.05) in their degree of the FFA release rate at 10 min, even though a slightly reduced FFA release rate was observed in the PHY emulsion during the lipolysis reaction. This suggests that the degree of CLn present at which position (i.e., sn-1 and sn-1,3) of the TAG molecules affects lipolysis. In particular, TAG consisting of three molecules of CLn (i.e., CLn-CLn-CLn) is considered to be the most important factor with respect to low lipolysis in this study. For example, PHY contained 24.8% CLn-CLn-CLn; however, PGO (showing the lowest lipolysis rate) consisted of 67.8% of CLn-CLn-CLn. In the pH-stat digest model, there was a lower initial rate of PHY than that of SBO (0% CLn-CLn-CLn) and IO (3.4% CLn-CLn-CLn at most). Nevertheless, the FFA release rate of PHY tended to be low with significant differences, at least up until 5 min of the lipolysis reaction. As expected, PGO containing the highest amount of CLn-CLn-CLn showed the lowest initial rate among them. Therefore, the low lipolysis characteristic of CLn-CLn-CLn may clearly be shown from PGO rather than from PHY. From the results, we conclude that the higher is the CLn-CLn-CLn content, the lower is the lipolysis rate; the order is as follows: SBO ≥ IO ≥ PHY > PGO. Such assumptions can be supported by the following experimental results that were derived by calculating the amount of residual TAG after lipolysis using HPLC-ELSD analysis (Equation (5)), in which CLn-CLn-CLn showed a lower lipolysis rate than any other TAG molecules (i.e., L-L-L, L-L-O, CLn-CLn-P, etc.). In particular, the proposed mathematical model for quantifying individual TAG species suggests that lipolysis rate of CLn-CLn-CLn in PGO sample was 65.3% while those of L-L-L and L-L-O present in SBO sample were 77.1% and 75.5%, respectively. However, there was no significant difference at *p* > 0.05 between CLn-CLn-CLn and L-L-L, although they were significant at *p* < 0.1. Since the digestive process in the human body is very complex and influenced by many factors, it is hard to say which factor most affects the rate of lipid digestion. Nevertheless, the results from this experiment can be used as a basis for understanding the effect of different compositional fatty acids in TAG molecules on lipid digestion.

## Figures and Tables

**Table 1:** Composition of duodenal and bile juices in the simulated small intestinal fluid.

	Duodenal Juice		Bile Juice	
Inorganic components	40 mL NaCl	175.3 g/L	30 mL NaCl	175.3 g/L
	40 mL NaHCO_3_	84.7 g/L	68.3 mL NaHCO_3_	84.7 g/L
	10 mL KH_2_PO_4_	8 g/L	4.2 mL KCl	89.6 g/L
	6.3 mL KCl	89.6 g/L		
	10 mL MgCl_2_	5 g/L		
	250 μL HCl 35%		350 μL HCl 35%	
Organic components	4 mL urea	25 g/L	10 mL urea	25 g/L
Mixture of organic and inorganic solution ^1^	9 mL CaCl_2_	16.8 g/L	10 mL CaCl_2_	16.8 g/L
	1 g BSA		1.8 g BSA	
			15 g bile salts ^2^	
pH	6.4 ± 0.2		6.4 ± 0.2	

^1^ Mixture of inorganic and organic solution was set up to 500 mL with distilled water. ^2^ The 15 g of bile salts were replaced by 30 g of bile extract in the simulated model of small intestinal digestion.

**Table 2:** Fatty acid compositions and profiles of plausible triacylglycerol species (peak area%).

**Fatty Acids**	**SBO ^1^**	**PGO**	**PHY**	**IO**
C16:0 (P, palmitic acid)	13.1 ± 0.1	4.0 ± 0.1	9.8 ± 0.3	8.8 ± 0.3
C18:0 (S, stearic acid)	3.8 ± 0.1	2.6 ± 0.0	4.0 ± 0.1	3.7 ± 0.1
C18:1 n-9 (O, oleic acid)	22.1 ± 0.2	7.0 ± 0.0	18.1 ± 0.6	16.7 ± 0.1
C18:1 n-7	2.8 ± 0.1	1.1 ± 0.1	2.4 ± 0.2	2.7 ± 0.4
C18:2t	0.5 ± 0.1	0.9 ± 0.0	0.6 ± 0.0	0.8 ± 0.1
C18:2 n-6 (L, linoleic acid)	49.5 ± 0.1	8.5 ± 0.3	35.1 ± 1.0	32.9 ± 0.2
C18:3 n-6 (γ-Ln, γ-linolenic acid)	0.6 ± 0.1	-	0.3 ± 0.0	0.3 ± 0.1
C18:3 n-3 (Ln, α-linolenic acid)	5.5 ± 0.2	-	3.4 ± 0.2	3.0 ± 0.1
C18:3 n-5 (CLn, conjugated linolenic acid)	-	74.7 ± 0.9	24.7 ± 2.0	29.2 ± 0.9
Unknown	2.0 ± 0.1	1.2 ± 0.3	1.8 ± 0.3	1.9 ± 0.1
**Plausible Major TAG Species (PN) ^2^**	**SBO**	**PGO**	**PHY**	**IO**
CLn-CLn-CLn (36)	-	67.8 ± 1.9	24.8 ± 1.1	-
CLn-CLn-CLn/CLn-CLn-Ln (36)	-	-	-	3.4 ± 0.4
CLn-CLn-L (38)	-	7.9 ± 0.8	3.0 ± 0.5	-
CLn-CLn-L/CLn-Ln-L (38)	-	-	-	18.0 ± 0.2
L-L-Ln (40)	6.0 ± 0.0	-	2.7 ± 0.5	-
L-L-Ln/L-L-CLn (40)	-	-	-	14.7 ± 0.3
CLn-CLn-O(P) (40)	-	12.1 ± 0.5	4.7 ± 1.2	-
CLn-CLn-O(P)/CLn-Ln-O(P) (40)	-	-	-	10.4 ± 0.4
L-L-L (42)	24.5 ± 0.1	-	12.9 ± 1.9	2.2 ± 0.1
O-L-Ln (42)	2.8 ± 0.1	-	1.7 ± 0.4	-
O-L-Ln/O-L-CLn (42)	-	-	-	18.5 ± 1.0
L-L-O (44)	21.4 ± 0.1	-	13.2 ± 0.2	5.2 ± 0.2
P-L-L (44)	15.2 ± 0.2	-	9.0 ± 0.3	5.9 ± 0.3
L-O-O (46)	6.8 ± 0.2	-	4.8 ± 0.2	1.4 ± 0.2
L-L-S/P-L-O (46)	12.7 ± 0.2	-	8.6 ± 0.1	3.0 ± 0.2
S-L-O (48)	4.2 ± 0.1	-	3.0 ± 0.5	-
Unknown	6.4 ± 0.4	12.2 ± 0.6	11.7 ± 1.2	17.2 ± 0.3

^1^ SBO, soybean oil; PGO, pomegranate seed oil; PHY, physical blend; IO, interesterified oil. ^2^ PN, partition number. All values are the mean ± standard deviation of two replicates.

## References

[1] Khoddami A., Che Man Y.B., Roberts T.H. (2014). Physico-chemical properties and fatty acid profile of seed oils from pomegranate (*Punica granatum* L.) extracted by cold pressing. Eur. J. Lipid Sci. Technol..

[2] Belitz H.D., Grosch W. (1999). Food Chemistry.

[3] Wang X.Y., Yang D., Gan L.J., Zhang H., Shin J.A., Park S.H., Lee K.T. (2014). Degree of oxidation depending on the positional distribution of linolenic acid in perilla oil and interesterified products. Food Sci. Biotechnol..

[4] Kohno H., Suzuki R., Yasui Y., Hosokawa M., Miyashita K., Tanaka T. (2004). Pomegranate seed oil rich in conjugated linolenic acid suppresses chemically induced colon carcinogenesis in rats. Cancer Sci..

[5] Lansky E.P., Newman R.A. (2007). *Punica granatum* (pomegranate) and its potential for prevention and treatment of inflammation and cancer. J. Ethnopharmacol..

[6] Sek L., Porter C.J.H., Kaukonen A.M., Charman W.N. (2002). Evaluation of the in-vitro digestion profiles of long and medium chain glycerides and the phase behaviour of their lipolytic products. J. Pharm. Pharmacol..

[7] Zhu X., Ye A., Verrier T., Singh H. (2013). Free fatty acid profiles of emulsified lipids during in vitro digestion with pancreatic lipase. Food Chem..

[8] Arishima T., Tachibana N., Kojima M., Takamatsu K., Imaizumi K. (2009). Screening of resistant triacylglycerols to the pancreatic lipase and their potentialities as a digestive retardant. J. Food Lipids.

[9] Martin J.C., Sébédio J.L., Caselli C., Pimont C., Martine L., Bernard A. (2000). Lymphatic delivery and in vitro pancreatic lipase hydrolysis of glycerol esters of conjugated linoleic acids in rats. J. Nutr..

[10] List G.R., Mounts T.L., Orthoefer F., Neff W.E. (1995). Margarine and shortening oils by interesterification of liquid and trisaturated triglycerides. J. Am. Oil Chem. Soc..

[11] Shimada Y., Sugihara A., Maruyama K., Nagao T., Nakayama S., Nakano H., Tominaga Y. (1996). Production of structured lipid containing docosahexaenoic and caprylic acids using immobilized *Rhizopus delemar* lipase. J. Ferment. Bioeng..

[12] Lee K.T., Akoh C.C. (1998). Structured lipids: Synthesis and applications. Food Rev. Int..

[13] Lee J.H., Lee K.T., Akoh C.C. (2006). Chapter 23, Structured Lipids Production. Handbook of Functional Lipids.

[14] Li Y., McClements D.J. (2010). New mathematical model for interpreting pH-stat digestion profiles: Impact of lipid droplet characteristics on in vitro digestibility. J. Agric. Food Chem..

[15] Jannin V., Dellera E., Chevrier S., Chavant Y., Voutsinas C., Bonferoni C., Demarne F. (2015). In vitro lipolysis tests on lipid nanoparticles: Comparison between lipase/co-lipase and pancreatic extract. Drug Dev. Ind. Pharm..

[16] Mat D.J.L., Le Feunteun S., Michon C., Souchon I. (2016). In vitro digestion of foods using pH-stat and the INFOGEST protocol: Impact of matrix structure on digestion kinetics of macronutrients, proteins and lipids. Food Res. Int..

[17] Giang T.M., Gaucel S., Brestaz P., Anton M., Meynier A., Trelea I.C., Feunteun S.Le. (2016). Dynamic modeling of in vitro lipid digestion: Individual fatty acid release and bioaccessibility kinetics. Food Chem..

[18] Christie W.W. (2003). Lipid Analysis.

[19] Adhikari P., Shin J.A., Lee J.H., Hu J.N., Hwang K.T., Lee K.T. (2009). Enzymatic production of *trans*-free hard fat stock from fractionated rice bran oil, fully hydrogenated soybean oil, and conjugated linoleic acid. J. Food Sci..

[20] Hur S.J., Decker E.A., McClements D.J. (2009). Influence of initial emulsifier type on microstructural changes occurring in emulsified lipids during in vitro digestion. Food Chem..

[21] Versantvoort C.H.M., Oomen A.G., Van de Kamp E., Rompelberg C.J.M., Sips A.J.A.M. (2005). Applicability of an in vitro digestion model in assessing the bioaccessibility of mycotoxins from food. Food Chem. Toxicol..

[22] Gan L.J., Yang D., Shin J.A., Kim S.J., Hong S.T., Lee J.H., Sung C.K., Lee K.T. (2012). Oxidative comparison of emulsion systems from fish oil-based structured lipid versus physically blended lipid with purple-fleshed sweet potato (*Ipomoea batatas* L.) extracts. J. Agric. Food Chem..

[23] Kaufman M., Wiesman Z. (2007). Pomegranate oil analysis with emphasis on MALDI-TOF/MS triacylglycerol fingerprinting. J. Agric. Food Chem..

[24] Mitra K., Lee J.H., Lee K.T., Kim S.A. (2010). Production tactic and physiochemical properties of low ω6/ω3 ratio structured lipid synthesised from perilla and soybean oil. Int. J. Food Sci. Technol..

[25] Armand M., Pasquier B., André M., Borel P., Senft M., Peyrot J., Salducci J., Portugal H., Jaussan V., Lairon D. (1999). Digestion and absorption of 2 fat emulsions with different droplet sizes in the human digestive tract. Am. J. Clin. Nutr..

[26] Bauer E., Jakob S., Mosenthin R. (2005). Principles of physiology of lipid digestion. Asian-Australas. J. Anim. Sci..

[27] Celia C., Trapasso E., Cosco D., Paolino D., Fresta M. (2009). Turbiscan Lab^®^ Expert analysis of the stability of ethosomes^®^ and ultradeformable liposomes containing a bilayer fluidizing agent. Colloids Surf. B Biointerfaces..

[28] Fioramonti S.A., Martinez M.J., Pilosof A.M.R., Rubiolo A.C., Santiago L.G. (2015). Multilayer emulsions as a strategy for linseed oil microencapsulation: Effect of pH and alginate concentration. Food Hydrocoll..

[29] Mengual O., Meunier G., Cayre I., Puech K., Snabre P. (1999). Characterisation of instability of concentrated dispersions by a new optical analyser: The TURBISCAN MA 1000. Colloids Surf. A Physicochem. Eng. Aspects.

[30] Golding M., Wooster T.J. (2010). The influence of emulsion structure and stability on lipid digestion. Curr. Opin. Colloid Interface Sci..

[31] Willis W.M., Lencki R.W., Marangoni A.G. (1998). Lipid modification strategies in the production of nutritionally functional fats and oils. Crit. Rev. Food Sci. Nutr..

[32] McClements D.J., Li Y. (2010). Review of in vitro digestion models for rapid screening of emulsion-based systems. Food Funct..

[33] Perona J.S., Ruiz-Gutierrez V. (2004). Quantification of major lipid classes in human triacylglycerol-rich lipoproteins by high-performance liquid chromatography with evaporative light-scattering detection. J. Sep. Sci..

[34] Moreau R.A., Lin J.T., McKeon T.A. (2005). The Evaporative Light-Scattering Detector as a Tool for the Analysis of Lipids by HPLC. HPLC of Acyl Lipids.

[35] Rodríguez-Alcalá L.M., Fontecha J. (2010). Major lipid classes separation of buttermilk, and cows, goats and ewes milk by high performance liquid chromatography with an evaporative light scattering detector focused on the phospholipid fraction. J. Chromatogr. A.

[36] Bottino N.R., Vandenburg G.A., Reiser R. (1967). Resistance of certain long-chain polyunsaturated fatty acids of marine oils to pancreatic lipase hydrolysis. Lipids.

[37] Brockerhoff H. (1970). Substrate specificity of pancreatic lipase. Influence of the structure of fatty acids on the reactivity of esters. Biochim. Biophys. Acta.

